# LMP1-Induced Cell Death May Contribute to the Emergency of Its Oncogenic Property

**DOI:** 10.1371/journal.pone.0060743

**Published:** 2013-04-23

**Authors:** Guillaume Brocqueville, Papa Alioune Ndour, Tan-Sothéa Ouk, Arnaud Le Goff, Caroline De Witte, Alexandra Mougel, Jean Coll, Véronique Fafeur, Xuefen Le Bourhis, Eric Adriaenssens

**Affiliations:** 1 CNRS UMR8161, Institut de Biologie de Lille, Institut Pasteur de Lille, IFR 142, Université Lille-Nord de France, Lille, France; 2 Laboratoire de Chimie des Substances Naturelles, SFR 145 GEIST, Université de Limoges, Limoges, France; 3 INSERM U908, Université Lille 1, Université Lille-Nord de France, Villeneuve d'Ascq, France; University of Hong Kong, Hong Kong

## Abstract

**Background and Objectives:**

Epstein-Barr Virus (EBV) Latent Membrane Protein 1 (LMP1) is linked to a variety of malignancies including Hodgkin's disease, lymphomas, nasopharyngeal and gastric carcinoma. LMP1 exerts its transforming or oncogenic activity mainly through the recruitment of intracellular adapters *via* LMP1 C-terminal Transformation Effector Sites (TES) 1 and 2. However, LMP1 is also reported to elicit significant cytotoxic effects in some other cell types. This cytotoxic effect is quite intriguing for an oncogenic protein, and it is unclear whether both functional aspects of the protein are related or mutually exclusive.

**Methodology and Principal Findings:**

Using different ectopic expression systems in both Madin-Darby canine kidney (MDCK) epithelial cells and human embryonic kidney HEK-293 cells, we observe that LMP1 ectopic expression massively induces cell death. Furthermore, we show that LMP1-induced cytotoxicity mainly implies LMP1 C-terminal transformation effector sites and TRADD recruitment. However, stable expression of LMP1 in the same cells, is found to be associated with an increase of cell survival and an acquisition of epithelial mesenchymal transition phenotype as evidenced by morphological modifications, increased cell mobility, increased expression of MMP9 and decreased expression of E-cadherin. Our results demonstrate for the first time that the cytotoxic and oncogenic effects of LMP1 are not mutually exclusive but may operate sequentially. We suggest that in a total cell population, cells resistant to LMP1-induced cytotoxicity are those that could take advantage of LMP1 oncogenic activity by integrating LMP1 signaling into the pre-existent signaling network. Our findings thus reconcile the apparent opposite apoptotic and oncogenic effects described for LMP1 and might reflect what actually happens on LMP1-induced cell transformation after EBV infection in patients.

## Introduction

Latent Membrane Protein 1 (LMP1) from Epstein-Barr virus (EBV) is thought to be the major oncogene accounting for most of EBV-related malignancies, including Burkitt lymphoma, Hodgkin disease, gastric carcinoma and nasopharyngeal carcinoma (NPC) [1]. This protein has been extensively demonstrated to transform B lymphocytes [2], T lymphocytes [3], monocytes [4] and fibroblasts [5].

LMP1 is a 63-kDa plasma membrane protein with a short N-terminal hydrophilic region, six transmembrane domains and a long C-terminal cytoplasmic region which is responsible for most of LMP1-induced biological effects. The latter is actually endowed with two critical signaling sites, named Transformation Effector Sites-1 and-2 (TES1 and TES2), that recruit a similar set of proximal intracellular adaptors as Tumor Necrosis Factor Receptor (TNFR) [6], [7], including TNFR-associated factors (TRAFs) and the TNFR-associated death domain protein (TRADD). LMP1 acts in a ligand-independent manner to activate several pathways including phosphatidylinositol 3-kinase (PI3K) [8], c-Jun N-terminal kinase (JNK) [9], p38 MAPK [10] and NF-κB [11], which lead to the expression of genes involved in cell survival, proliferation and migration [12], [13], [14].

However, several studies have reported that LMP1 also exerts cytotoxic properties. For instance, high levels of LMP1 have been described to be toxic [15]. Moreover, LMP1 is able to trigger cell death in an NF-κB-dependent manner [16]. The pro-apototic effect of LMP1 has been observed in various cell types, including lymphoblastoid cell lines [17], [18], [19], monocytes [20] and epithelial cells [21], [22], [23].

Although the antagonistic actions of LMP1 have been observed in different cell types, it is unclear if the both actions are mutually exclusive or functionally related in the context of a heterogeneous cellular population. The conclusions of a study performed by Kim and colleagues [24], and our own previous observations provide a first clue. On one hand, the formers have shown that MDCK cells become able to scatter and form tubules as a result of stable LMP1 expression, both phenotypes being related to LMP1 oncogenic properties. However, when we tried to establish MDCK cells stably expressing LMP1 by transfecting cells with LMP1-expressing vectors, we found that the majority of cells were committed to death. This work was thus designed to better clarify the apparent opposite cytotoxic and oncogenic effects of LMP1 in the context of cancer development. We observed that massive cell death before the establishment of cells stably expressing LMP1 is a common process. We suggest that in a total cell population, cells resistant to LMP1-induced cytotoxicity are those that could take advantage of LMP1 oncogenic activity by integrating LMP1 signaling into the pre-existent signaling network. Our findings thus reconcile the apparent opposite apoptotic and oncogenic effects described for LMP1 and might reflect what actually happens on LMP1-induced cell transformation after EBV infection in patients.

## Results and Discussion

### LMP1 induces cell death in MDCK epithelial cells

**Figure 1:**
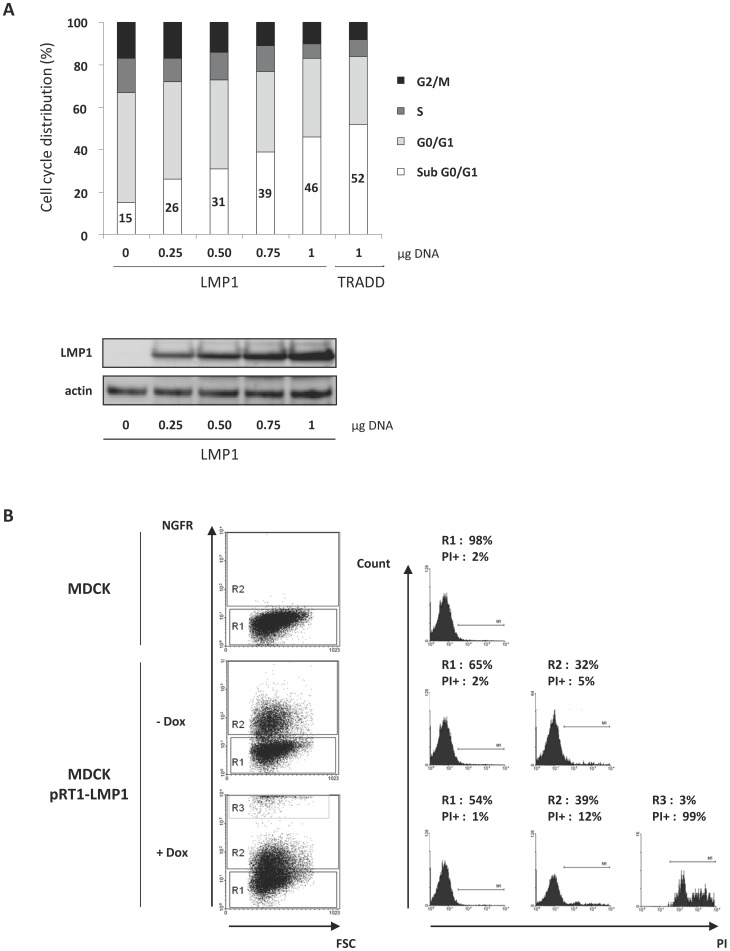
Ectopic expression of LMP1 in MDCK cells induced cell death. (**A**) MDCK cells were transfected with increasing amounts (0–1 µg) of LMP1 vector. Cells were then permeabilized with ethanol and labelled with propidium iodide (PI). Cell cycle distribution was analyzed using flow cytometry. TRADD-expressing cells were used as a control of cell death induction. LMP1 expression was analyzed by Western blot. Actin was used as a loading control. (**B**) MDCK cells were transfected with a doxycyline (Dox)-inducible vector (pRT1-LMP1) endowed with a bidirectional promoter which drives the expression of both LMP1 and a truncated version of NGF receptor (NGFRt) serving as a reporter gene. The detection of NGFRt at the surface of cells implies that these cells also express LMP1. Cells were treated (+Dox) or not (- Dox) with Dox (2 µg/ml) for 24 hours, stained with PI and PE-conjugated antibodies directed against NGFRt, and then analyzed by flow cytometry. Percentages of cells in each cell population (R1: negative NGFRt labelling; R2: low NGFRt labelling; R3: high NGFRt labelling) and corresponding propidium iodide positive cells (PI+) (i.e dead cells) are indicated. Parental MDCK cells were used as a control.

To assess the impact of LMP1 expression, we transiently transfected MDCK cells with increasing amounts of a vector encoding LMP1 (but equal amounts of DNA in total), and then performed cell cycle analysis by flow cytometry (Fig 1A). The percentage of cells in sub G0/G1 (loss of DNA content) is indicative of dead cells. Empty vector transfected cells exhibited 15% of cell death, LMP1 induced cell death in a dose-dependent manner, attaining almost half of the cell population when 1 µg of vector encoding LMP1 cDNA was used. MDCK cells ectopically expressing the death-inducing TRADD protein were used as a positive control.

To further confirm the ability of LMP1 to evoke cell death, MDCK cells were stably transfected with a bidirectional, Tet-On inducible plasmid encoding a truncated version of NGF receptor (NGFR) and LMP1. As NGFR-expressing cells inevitably express LMP1, NGFR expression is used here for the tracking of LMP1-expressing cells. Cells were treated for 24 h with doxycycline to induce gene expression, labeled with both anti-NGFR antibodies and propidium iodide, and then analyzed using flow cytometry to assess cell death. As expected, non transfected parental MDCK cells did not express NGFR and only 2% of them were positive for PI staining (i.e dead) (Fig 1B). Surprisingly, in the absence of doxycycline, 32% of transfected cells were NGFR-positive (R2), meaning that LMP1 was also expressed in these cells. However, this leak of LMP1 expression was associated with a slightly increased proportion of dead cells (from 2% to 5%), indicating that the majority of LMP1-expressing cells were alive. Doxycycline treatment induced an increase of LMP1 expression as revealed by the appearance of a subpopulation expressing high levels of LMP1 (R3) and an increase of cells expressing more moderate levels of LMP1 (R2). Contrary to results obtained in the absence of doxycyclin induction, it seemed that almost all the cells induced to express LMP1 finished by dying: an increase of moderately expressing LMP1 cells (from 32% to 39%) was correlated with the same increase of dead cells (from 5% to 12%); most significant was the dramatic death rate (99%) for cells expressing very high levels of LMP1 (R3).

Therefore, using the both transient and stable expression systems, we found that LMP1 was cytotoxic to MDCK cells when expressed at relatively high levels. Moreover, the cytotoxic effect was associated with levels of its expression. Thus, even in MDCK cells, which have been reported to be transformed by LMP1 [24], LMP1 can exert also a cytotoxic action.

### The C-terminal tail is involved in LMP1-induced cell death

**Figure 2:**
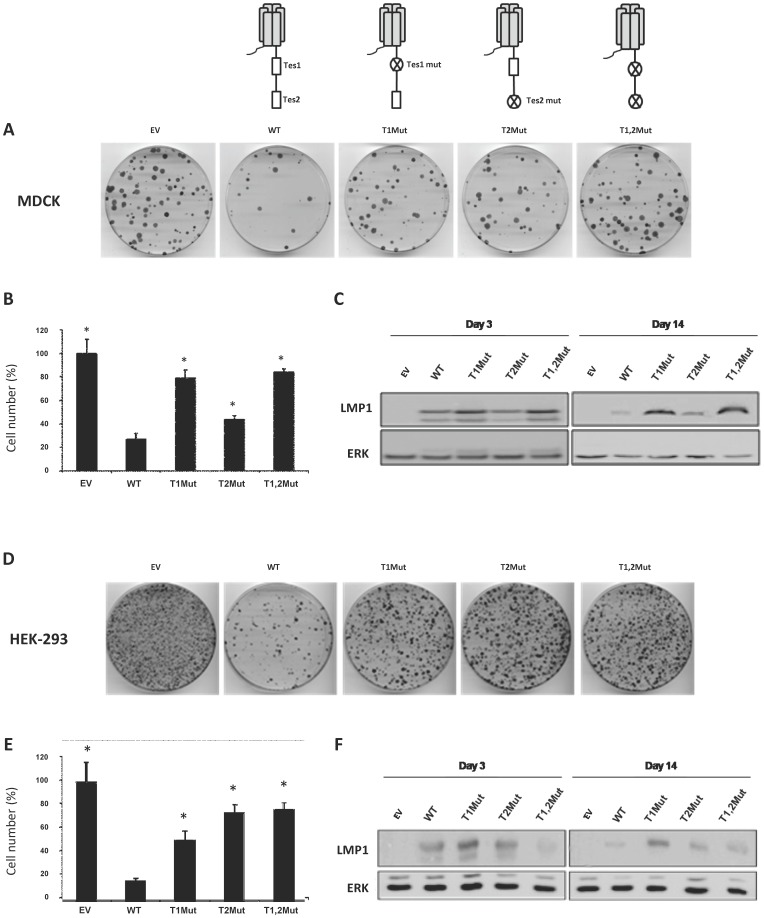
LMP1 induced cell death through its C-terminal tail. MDCK (**A to C**) and HEK-293 (**D to F**) cells were transfected with a empty vector (-) or a vector driving the expression of different versions of LMP1: the wild-type protein (WT) and versions of LMP1 in which either TES1 (T1Mut), or TES2 (T2Mut) or both (T1,2Mut) are altered. Two days later, cells were split into 5 dishes and cultured for 11 days in the presence of G418 to favor the selection of transfected cells. (**A**, **D**) Pictures of cells stained with Giemsa. (**B**, **E**) Cell counting. Results are represented as the mean of three different dishes, and given as a percentage of cells transfected with the empty plasmid (100%). Standard deviations are shown. *p-value of <0.05, compared to the WT LMP1 expressing vector (WT), two-sample *t* test (two-tailed). (**C**, **F**) Whole cell extracts from cells cultured for 3 days or 11 days in the presence of G418 were prepared. Proteins were resolved by 10% SDS-PAGE and analyzed by Western blot using antibodies directed against LMP1 or ERK2 (used as a loading control).

LMP1 contains two Transformation Effector Sites (TES1 and TES2) that are able to recruit different proteins known to initiate various signaling pathways [6]. Therefore, these regions of the protein are considered to account for most of LMP1-associated biological responses. To assess whether or not TES1 and/or TES2 are critical in LMP1-induced cell death, MDCK normal epithelial cells and and human embryonic kidney HEK-293 cells were transfected with vectors expressing different versions of LMP1: the wild-type protein (WT) or modified versions in which either TES1 (T1Mut), TES2 (T2Mut) or both (T1, 2Mut) are altered. Cells were then cultured in presence of G418. As the vector also encodes the gene (*neo*) that confers resistance against G418, only cells efficiently transfected are expected to survive the treatment, unless they ectopically express a cytotoxic product. As shown in Fig 2, WT LMP1-transfected cells formed less clones and exhibited a strong decrease of cell number when compared to empty vector-transfected cells. Disruption of TES1 or TES2 partially restored clone formation and cell growth, indicating that both TES1 and TES2 are involved in LMP1-induced cytotoxicity. However, this meaned also that other domains of the protein might be required. Accordingly, it has been previously reported that the transmembrane domains can exert cytostatic activities [25]. Interestingly, when protein expression was analysed using Western blot (Fig 2C and F), we observed that after two weeks of antibiotic selection, WT LMP1 levels were strongly reduced compared to the other variants. This indicated that selected cells were not only resistant to antibiotic but also expressed a low level of LMP1, further confirming the cytoxicity of WT LMP1 when expressed at high level. Of note, LMP1 cytotoxic effect was also observed in cancer cells including Hela (data not shown) and Jurkat (Fig S1 and File S1) cell lines.

The clonogenic assay also revealed that both TES1 and TES2 are involved in mediating colony formation, with a stronger involvement of the TES1 domain in MDCK cells and of the TES2 domain in HEK293 cells. It is possible that at least one signaling pathway induced by these two domains contributes both to cell death and colony formation. This discrepancy could be due to differential adapter protein equipment in these two cell lines.

### LMP1-induced cell death implies the recruitment of TRADD

**Figure 3:**
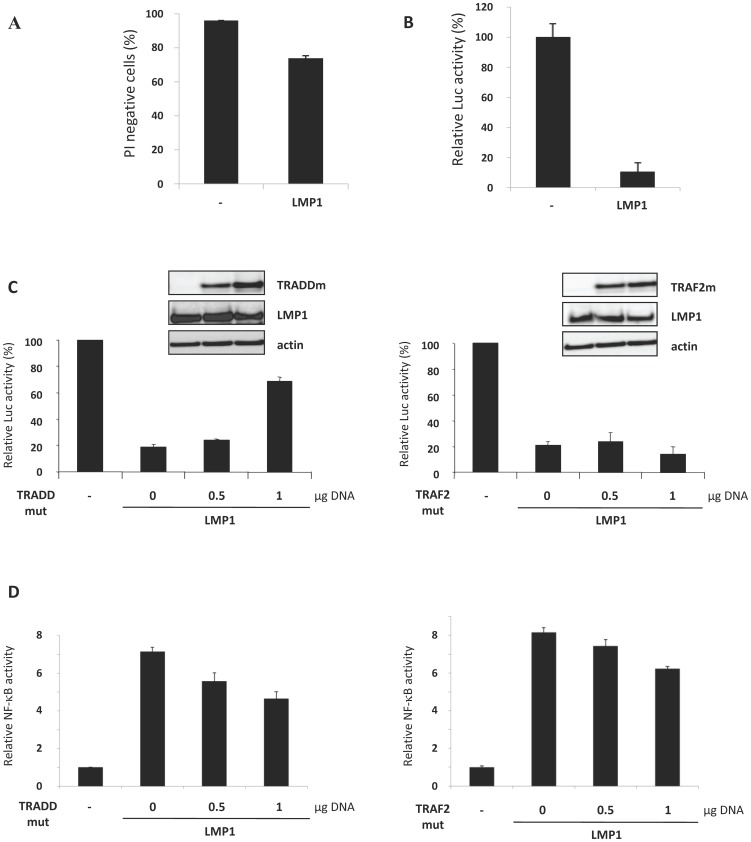
TRADD was involved in LMP1-induced cell death. (**A**) MDCK cells were transfected with an empty vector or a vector expressing LMP1. Cells were then incubated with propidium iodide (PI) before analysis of PI labelling using flow cytometry. (**B**) MDCK cells were co-transfected with pRLnull and LMP1 plasmids. Cell viability was determined by a luciferase activity assay. Luciferase activity of cells transfected with empty vectors was indexed to 100%. Values represent the means of triplicate assays ± SD from a representative experiment. Experiments were performed three times and similar results were obtained. (**C**) MDCK cells were co-transfected with the pRL null plasmid, the LMP1 expressing plasmid (1 µg DNA) and increasing doses (0, 0.5, 1 µg DNA) of plasmids expressing dominant negative versions of TRADD protein (TRADD mut) or TRAF2 (TRAF2 mut). Cell viability was determined by assessing the activity of Renilla luciferase. The luciferase activity of control cells transfected with empty vectors was indexed to 100%. Values represent the means of triplicate assays ± SD of a representative experiment. Experiments were performed three times and similar results were obtained. Whole cell extracts were resolved by 10% SDS-PAGE and analyzed by Western blot to assess gene expression, using antibodies directed against LMP1, TRADD or TRAF2. Actin was used as a loading control. (**D**) Cells were co-transfected with a vector in which five elements responding to NFκB control the expression of the firefly luciferase gene, a LMP1 expressing vector and vectors expressing dominant negative versions of TRADD protein (TRADD mut) or TRAF2 (TRAF2 mut). The luciferase activity of control cells transfected with empty vectors was indexed to 1.

TES1 and TES2 are known to bind the same proximal adaptors as the TNF Receptor (TNFR) [6], [7], including TRAFs and TRADD, which results in the activation of signaling pathways such as NF-κB [11]. We then investigated which TES-associated adaptors could be recruited in the cytotoxic context and convey the signal to cell death. For this, we co-transfected MDCK cells with a vector expressing LMP1, vectors expressing dominant negative versions of either TRADD (TRADDmut) or TRAF2 (TRAF2mut) and a vector encoding the *Renilla* luciferase. As the normalizing pRLnull luciferase reporter vector does not contain any promoter or enhancer element, the basal luciferase activity reflects only transfected living cells, whatever cellular context. This reporter system was thus used to overcome the limit of cell death detection by direct IP staining, which did not allow us to distinguish transfected cells from non-transfected ones (Fig 3A). As shown in Figure 1B, massive cell death was induced by LMP1 in MDCK cells, since less than 20% of cells expressed luciferase. TRADDmut (Fig 3C) decreased LMP1-induced cell death in a dose-dependent manner, with an increase of about 40% of viable cells compared to those expressing only LMP1. On the contrary, the co-expression of TRAF2mut did not affect cell viability. These constructs are functional, as revealed by their ability to decrease NF-κB activation (Fig 3D). The partial inhibitory effect may be explained by the fact that other adaptors are also involved in the activation of NF-κB. TRADD and TRAF2 are key adaptors of TNFR-associated biological responses and are known to promote either survival or cell death depending on the cellular context. Our data showed that TRADD, but not TRAF2, is involved in LMP1-mediated cytotoxicity.

### Stably LMP1 expressing MDCK cells exhibit epithelial mesenchymal transition phenotype and enhanced survival

**Figure 4:**
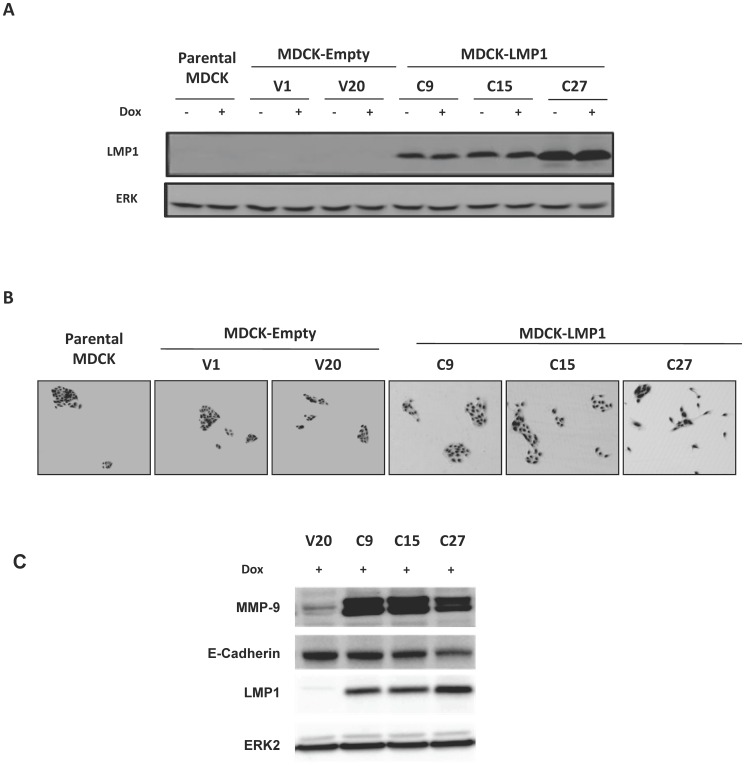
Characterization of MDCK cell lines stably expressing LMP1. MDCK cells were transfected with the pSTAR encoding or not LMP1 cDNA After selection with G418, two control clones (V1 and V20) and three LMP1-expressing clones (C9, C15 and C27) were selected and further analyzed with (Dox+) or without (Dox-) doxycycline induction. (**A**) Expression of LMP1 by Western blot analysis. ERK was used as a loading control. (**B**) Representative pictures of each cell line. Parental MDCK cells are shown as a reference. (**C**) Expression of LMP1, E-cadherin and MMP9. ERK was used as a loading control.

Although LMP1 clearly exerted cytotoxic effect in different cell types, cells expressing low to moderate amounts of the protein could still survive (Fig 1B; Fig 2). We tried to generate stable MDCK cell lines using a vector (pSTAR) allowing inducible gene expression by doxycycline treatment [26]. The induction system was chosen to allow for the study of clones suffering from LMP1 cytotoxic effects as well as clones able to express LMP1 without dying. Although the used plasmid was designed to prevent transgene expression before any doxycycline supplementation (i.e a similar number of clones was expected to emerge whether cells contained the transgene or not), only a few clones carrying it went through the antibiotic selection and could be further characterized for LMP1 expression (clones C9, C15 and C27) (Fig 4A). Two control clones (empty pSTAR, clones V1 and V20) were also analyzed. Surprisingly, doxycycline treatment did not increase LMP1 expression in the three selected clones (Fig 4A). However, this expression system has been successfully used by us to express adaptor signaling proteins such as IκBα, STAT1α, STAT1β and LMP1-DN [18], [27]. Herein, it is possible that induction of LMP1 expression might rapidly and massively induce death of cells without expression leak, thus eliminating any LMP1 overexpressing cells. Meanwhile, low or moderate levels of LMP1 due to expression leak in a subpopulation of cells could allow them to avoid death; further increase of LMP1 levels upon doxycycline induction should become fatal for these cells, as evidenced in Fig 1B. The final outcome of such a selection is the elimination of inducible clones with or without expression leak of LMP1.

**Figure 5:**
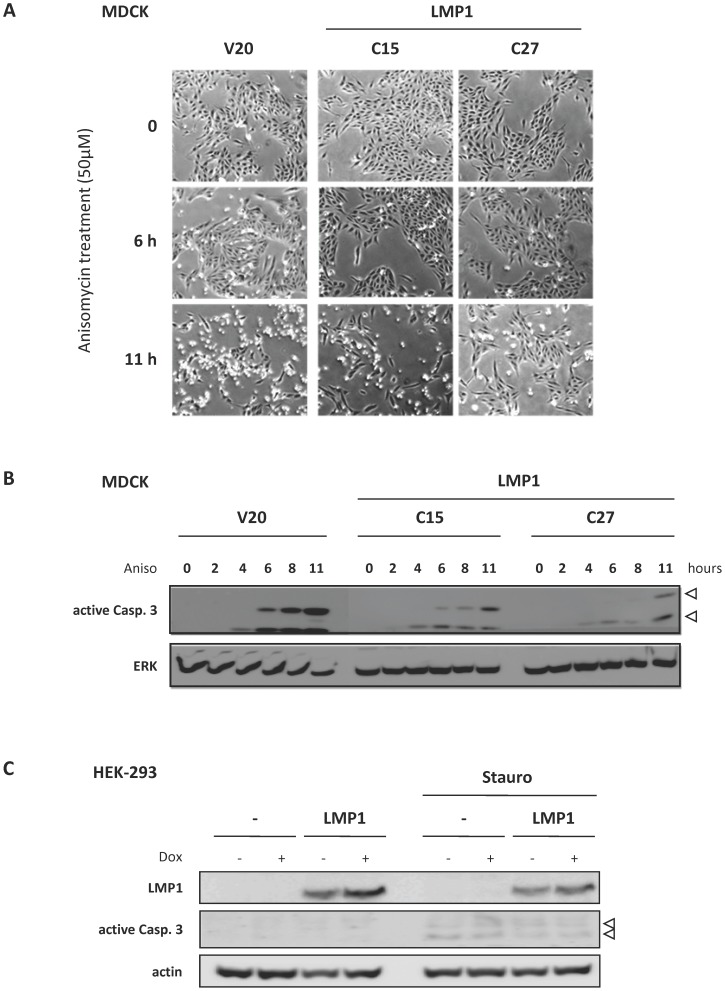
Cell lines stably expressing LMP1 were more resistant to stress-induced cell death. (**A**, **B**) MDCK cell lines stably expressing LMP1 (clones C15 and C27) and a control clone (V20) were treated with anisomycin (Aniso), an inhibitor of translation known to induce apoptose. (**A**) Representative pictures of cells cultured in the presence or absence of anysomycin (light microscope, magnification x20). (**B**) Detection of caspase 3 activation by Western blot analysis. Whole cell extracts were prepared after different periods of anysomycin treatment. Proteins were then resolved by 10% SDS-PAGE before Western blot analysis using antibodies against cleaved (i.e active form) caspase 3. ERK2 detection was used as loading control. (**C**) HEK-293 cell line stably expressing LMP1 and a control clone were treated for 8 hours with staurosporin (Stauro, 3 µM). Whole cell extracts were prepared and proteins were resolved by 10% SDS-PAGE before Western blot analysis of cleaved caspase 3 (white arrow). Actin was used as loading control.

Importantly, we found that cells stably expressing LMP1 were more scattered compared to control cells (Fig 4B). This phenomenon was accompanied by an increased expression of MMP9 and a decreased expression of E-cadherin (Fig 4C), indicating the acquisition of epithelial mesenchymal transition phenotype. Our findings are in agreement with the well described transforming ability of LMP1 in various cell types including MDCK cells [24], [28], [29]. We then determined the resistance of LMP1 expressing cells to anisomycin stress, an inhibitor of protein synthesis known to induce apoptosis. Compared to control cells (V20), LMP1 expressing clones (C15 and C27) were less detached from culture dishes (Fig 5A) and exhibited lower caspase 3 activation (Fig 5B). In addition, C27 clone expressing higher levels of LMP1 was more resistant to anisomycin-induced cell detachment and caspase 3 activation compared to C15 clone. Using another cell model HEK-293, we confirmed the anti-apoptotic effect of LMP1, as stable LMP1 expression in these cells reduced caspase 3 activation by staurosporin (Fig 5C). These results indicated that LMP1 could protect cells from cell death induction, which is ultimately consistent with the biological properties usually described for LMP1 as an oncogene [2], [7].

## Conclusion

LMP1 is the major oncogene of Epstein-Barr Virus and is found expressed in most EBV-associated malignancies. This protein has indeed been shown to transform B lymphocytes, T lymphocytes, monocytes, fibroblasts and also epithelial cells. Paradoxically, LMP1 has also been reported to promote or sensitize cells to apoptosis in various cell types, such as monocytes, lymphoblastoid cell lines and epithelial cells. Thus, LMP1 is interestingly endowed with two distinct, opposed facets depending on the cellular context in which the protein is expressed. In this work, we found that ectopic expression of LMP1 massively induced cell death. LMP1-induced cytotoxicity implied LMP1 C-terminal Transformation Effector Sites and TRADD recruitment. After the cytotoxic crisis, LMP1 expressing cells exhibited transforming characteristics including acquisition of epithelial mesenchymal transition phenotype and increased resistance to cell death. This massive cell death before the establishment of cells stably expressing LMP1 seemed to be a common process whatever cell types. We suggest that in a total population, cells resistant to LMP1-induced cell death are those that could take advantage of LMP1 oncogenic activity by integrating LMP1 signaling into the pre-existent signaling network. Our findings reconcile the apparent opposite apoptotic and oncogenic effects described for LMP1 and might reflect what actually happens on LMP1-induced cell transformation after EBV infection in patients.

## Methods

### Cytokines, drugs and cell cultures

Anysomycin was purchased from Calbiochem. Both doxycycline and staurosporine were purchased from Sigma. Hygromycin B was purchased from Euromedex. Geneticin (G418) was purchased from Invitrogen. Madin-Darby Canine Kidney (MDCK, CCL-34) epithelial cells and Human Embryonic Kidney 293 (HEK-293, CRL-1573) from ATCC and were grown in Dulbecco's modified Eagle's medium (DMEM, Invitrogen) supplemented with decomplemented fetal bovine serum (FBS, 10% v/v, Invitrogen) and antibiotics. Cells were cultured at 37°C in water-saturated, 5% CO_2_ atmosphere.

### Plasmids

pcDNA3-LMP1 expression vector was generated by inserting wt LMP1 cDNA, derived from the B95.8 strain of EBV into the empty pcDNA3 vector. pcDNA3-LMP1 mutants (named T1Mut, T2Mut and T1,2Mut) were generated by site-directed mutagenesis as previously described [30], [31]. Basically, to generate T1Mut, codons encoding aminoacyl residues PxQxT (204–208) were substituted with codons encoding residues AxAxA in LMP1 cDNA. To generate T2Mut, codons encoding residues YYD (384–386) were deleted. The variant form T1,2Mut carries both mutations. The inducible pRT1-LMP1 vector was a generous gift of G. W. Bornkamm [32]. The tetracycline-inducible promoter drives the bidirectional expression of both LMP1 and an inactive truncated version of NGFR lacking the cytoplasmic domain (NGFRt). pSTAR-LMP1 plasmid was generated by inserting wt LMP1 cDNA, derived from the B95.8 strain of EBV into the pSTAR empty vector [26]. The inducible JF_2_A-LMP1 vector, used to generate stable expression of LMP1 in HEK-293 cells, was a generous gift of J. Feuillard, Limoges, France.

TRADD and TRADD-DN expression vector were a generous gift of M. Rothe (Tularik Inc.). TRAF2-DN expression plasmid was a generous gift of P. Mehlen (Lyon, France). The normalizing vector pRLnull has no promoter sequence to drive expression of the Renilla luciferase gene and was purchased from Promega. The κB luciferase reporter construct has five NF-κB-responsive elements in tandem and was purchased from Stratagene.

### Antibodies

Anti-LMP1 monoclonal antibody was produced from a S12 hybridoma culture supernatant (a generous gift of P. Busson, Paris, France). Anti-actin (sc-47778), anti-ERK2 (sc-154), anti-TRADD (sc-1163), anti-TRAF2 (sc-876), anti-E-cadherin (sc-7870) antibodies (dilution 1/1000) were purchased from Santa Cruz Biotechnology. Anti-cleaved caspase 3 (9664) antibody was purchased from Cell Signaling Technology. Anti-MMP-9 (#1939-1) antibody was purchased from Epitomics. Anti-NGFR (nerve growth factor receptor), phycoerythrin (PE)- conjugated monoclonal antibody (C40-1457) were purchased from BD Pharmingen.

### Transfections

For transient expression experiments, MDCK cells (9×10^4^ cells/well) or HEK-293 (3×10^5^ cells/well) were seeded in six-well dishes. Transfections were performed using Lipofectamine™ 2000 Transfection Reagent (Invitrogen). For luciferase activity assay, 100 ng of κB luciferase reporter construct and 25 ng of normalizing vector pRLnull was cotransfected with 1–2 µg of DNA of interest. For dose effect studies, the amount of DNA was completed with the empty vector. Cells were incubated with the reagents for 5 hours at 37°C before the culture medium was replaced with fresh one supplemented with serum for further culture.

For stable expression experiments involving the inducible pRT1-LMP1 or JF_2_A-LMP1 vectors, MDCK or HEK-293 cells were transfected as described above. Two days after transfection, cells were cultured in DMEM supplemented with tetracycline-free FBS (10% v/v) and hygromycin B (150 µg/ml) for 3–4 weeks for the selection. LMP1 expression in selected clones was induced by doxycycline (2 µg/ml).

For stable expression experiments involving the pSTAR vectors, MDCK cells were transfected as described above with either the empty vector or the pSTAR-LMP1 vector. After 2 days, cells were further cultured in DMEM supplemented with tetracycline-free FBS (10% v/v) and G418 (800 µg/ml) for 2 weeks to establish stable clones.

### Luciferase activity assay

To measure the reporter activity, cells were harvested 24 hours after transfection, with a passive lysis buffer (Promega) and lysates were clarified by centrifugation. Luciferase assay was performed using a Luciferase assay system (Promega) and Luciferase activities were measured using a lumat LB 9501 (Berthold). Triplicate samples were performed in each experiment, and standard deviations (s.d.) are shown.

### Western blotting

Cells were lysed in PY lysis buffer (20 mM Tris-HCl pH 7.4, 50 mM NaCl, 5 mM EDTA, 1% Triton X-100) and western blotting was performed, as previously described [27].

### Measurement of cell death using flow cytometry analysis

Cell death was assessed using flow-cytrometry analysis after propidium iodide (PI, Sigma) labelling as previously described [33]. For cell cycle analysis, cells were washed with ice-cold PBS, and then incubated with ice-cold 70% ethanol. Fixed cells were washed and then treated with RNase A (1 mg/ml) and stained with PI (100 µg/ml). The percentage of cells in each phase of cell cycle (i.e G0/G1, S, G2M) as well as the percentage of dead cells (sub G0/G1) were then determined by measurement of DNA content using a flow cytometer (Coulter EPICS XL-MCL).

For immunofluorescent labelling of NGFR, cells were washed and incubated with PE-conjugated NGFR antibody for 30 min at 37°C. Cells were then analyzed using a Coulter EPICS XL-MCL flow cytometer and the data were analyzed using the WinMDI 2.9 software.

### Colony-formation assay

MDCK (10^5^ cells/well) and HEK-293 (3.10^5^ cells/well) were seeded in six-well dishes and transfected with 1 µg DNA per well using Lipofectamine 2000 as described above. Two days later, one part of cells was lyzed for western blot analysis, another part of cells was split into five 100 mm dishes and further cultured in DMEM supplemented with FBS (10%, v/v). The following day, the medium was supplemented with G418 (800 µg/ml) and cells were cultured in G418 containing medium for 11 days. Cells from one of the five dishes were stained (Giemsa, Sigma), while cells from another dish were lyzed for western blot analysis. The three other dishes were used for cell counting.

## Supporting Information

Figure S1 **Ectopic expression of LMP1 in Jurkat cells induced both cell death and cell survival.** **A**) Cell cycle analysis was performed after transient LMP1 transfection and cycloheximide treatment. Cells were transfected with empty vector as control or LMP1 encoding vector. After 24 h, cells were treated with the indicated doses of cycloheximide during 24 h and cell cycle was analyzed by flow cytometry. Percentage of sub G0/G1 cells was indicated. **B**) Number of clones obtained after stable transfection of LMP1-derived plasmids. After transfection, selection was added and cells were dispatched in 96-well plates. **C**) LMP1 protects Jurkat cells during serum deprivation or after UV irradiation. Stably transfected cells were starved during 24 h or treated by UV and cell death was monitored after annexin V and propidium iodine labeling. See File S1 for information.
(TIF)

File S1 (DOCX)

## References

[1] Rickinson AB, Kieff K (2007) Epstein-Barr Virus. In: Knipe DM, Howley, P M, editor. Fields Virology. Philadelphia: Lippincott-Raven. pp. 2655–2700.

[2] Kaye KM, Izumi KM, Kieff E (1993) Epstein-Barr virus latent membrane protein 1 is essential for B-lymphocyte growth transformation. Proc Natl Acad Sci U S A 90: 9150–9154. 8415670 10.1073/pnas.90.19.9150 PMC47519

[3] Groux H, Cottrez F, Montpellier C, Quatannens B, Coll J, et al (1997) Isolation and characterization of transformed human T-cell lines infected by Epstein-Barr virus. Blood 89: 4521–4530. 9192776

[4] Masy E, Adriaenssens E, Montpellier C, Crepieux P, Mougel A, et al (2002) Human monocytic cell lines transformed in vitro by Epstein-Barr virus display a type II latency and LMP-1-dependent proliferation. J Virol 76: 6460–6472. 12050358 10.1128/JVI.76.13.6460-6472.2002 PMC136267

[5] Roberts ML, Cooper NR (1998) Activation of a ras-MAPK-dependent pathway by Epstein-Barr virus latent membrane protein 1 is essential for cellular transformation. Virology 240: 93–99. 9448693 10.1006/viro.1997.8901

[6] Eliopoulos AG, Young LS (2001) LMP1 structure and signal transduction. Semin Cancer Biol 11: 435–444. 11669605 10.1006/scbi.2001.0410

[7] Gires O, Zimber-Strobl U, Gonnella R, Ueffing M, Marschall G, et al (1997) Latent membrane protein 1 of Epstein-Barr virus mimics a constitutively active receptor molecule. Embo J 16: 6131–6140. 9359753 10.1093/emboj/16.20.6131 PMC1326297

[8] Dawson CW, Tramountanis G, Eliopoulos AG, Young LS (2003) Epstein-Barr virus latent membrane protein 1 (LMP1) activates the phosphatidylinositol 3-kinase/Akt pathway to promote cell survival and induce actin filament remodeling. J Biol Chem 278: 3694–3704. 12446712 10.1074/jbc.M209840200

[9] Eliopoulos AG, Young LS (1998) Activation of the cJun N-terminal kinase (JNK) pathway by the Epstein-Barr virus-encoded latent membrane protein 1 (LMP1). Oncogene 16: 1731–1742. 9582021 10.1038/sj.onc.1201694

[10] Eliopoulos AG, Gallagher NJ, Blake SM, Dawson CW, Young LS (1999) Activation of the p38 mitogen-activated protein kinase pathway by Epstein-Barr virus-encoded latent membrane protein 1 coregulates interleukin-6 and interleukin-8 production. J Biol Chem 274: 16085–16096. 10347160 10.1074/jbc.274.23.16085

[11] Paine E, Scheinman RI, Baldwin AS Jr, Raab-Traub N (1995) Expression of LMP1 in epithelial cells leads to the activation of a select subset of NF-kappa B/Rel family proteins. J Virol 69: 4572–4576. 7769726 10.1128/jvi.69.7.4572-4576.1995 PMC189208

[12] Dawson CW, Laverick L, Morris MA, Tramoutanis G, Young LS (2008) Epstein-Barr virus-encoded LMP1 regulates epithelial cell motility and invasion via the ERK-MAPK pathway. J Virol 82: 3654–3664. 18199641 10.1128/JVI.01888-07 PMC2268486

[13] Everly DN Jr, Mainou BA, Raab-Traub N (2004) Induction of Id1 and Id3 by latent membrane protein 1 of Epstein-Barr virus and regulation of p27/Kip and cyclin-dependent kinase 2 in rodent fibroblast transformation. J Virol 78: 13470–13478. 15564458 10.1128/JVI.78.24.13470-13478.2004 PMC533955

[14] Shair KH, Schnegg CI, Raab-Traub N (2008) EBV latent membrane protein 1 effects on plakoglobin, cell growth, and migration. Cancer Res 68: 6997–7005. 18757414 10.1158/0008-5472.CAN-08-1178 PMC2593097

[15] Hammerschmidt W, Sugden B, Baichwal VR (1989) The transforming domain alone of the latent membrane protein of Epstein-Barr virus is toxic to cells when expressed at high levels. J Virol 63: 2469–2475. 2542565 10.1128/jvi.63.6.2469-2475.1989 PMC250704

[16] Nitta T, Chiba A, Yamashita A, Rowe M, Israel A, et al (2003) NF-kappaB is required for cell death induction by latent membrane protein 1 of Epstein-Barr virus. Cell Signal 15: 423–433. 12618217 10.1016/s0898-6568(02)00141-9

[17] Le Clorennec C, Ouk TS, Youlyouz-Marfak I, Panteix S, Martin CC, et al (2008) Molecular basis of cytotoxicity of Epstein-Barr virus (EBV) latent membrane protein 1 (LMP1) in EBV latency III B cells: LMP1 induces type II ligand-independent autoactivation of CD95/Fas with caspase 8-mediated apoptosis. J Virol 82: 6721–6733. 18448526 10.1128/JVI.02250-07 PMC2447067

[18] Le Clorennec C, Youlyouz-Marfak I, Adriaenssens E, Coll J, Bornkamm GW, et al (2006) EBV latency III immortalization program sensitizes B cells to induction of CD95-mediated apoptosis via LMP1: role of NF-kappaB, STAT1, and p53. Blood 107: 2070–2078. 16317104 10.1182/blood-2005-05-2053

[19] Zou P, Kawada J, Pesnicak L, Cohen JI (2007) Bortezomib induces apoptosis of Epstein-Barr virus (EBV)-transformed B cells and prolongs survival of mice inoculated with EBV-transformed B cells. J Virol 81: 10029–10036. 17626072 10.1128/JVI.02241-06 PMC2045383

[20] Li L, Liu D, Hutt-Fletcher L, Morgan A, Masucci MG, et al (2002) Epstein-Barr virus inhibits the development of dendritic cells by promoting apoptosis of their monocyte precursors in the presence of granulocyte macrophage-colony-stimulating factor and interleukin-4. Blood 99: 3725–3734. 11986229 10.1182/blood.v99.10.3725

[21] Lu JJ, Chen JY, Hsu TY, Yu WC, Su IJ, et al (1996) Induction of apoptosis in epithelial cells by Epstein-Barr virus latent membrane protein 1. J Gen Virol 77: 1883–1892. 8760440 10.1099/0022-1317-77-8-1883

[22] Zhang X, Hu L, Fadeel B, Ernberg IT (2002) Apoptosis modulation of Epstein-Barr virus-encoded latent membrane protein 1 in the epithelial cell line HeLa is stimulus-dependent. Virology 304: 330–341. 12504573 10.1006/viro.2002.1640

[23] Zhang X, Sanmun D, Hu L, Fadeel B, Ernberg I (2007) Epstein-Barr virus-encoded LMP1 promotes cisplatin-induced caspase activation through JNK and NF-kappaB signaling pathways. Biochem Biophys Res Commun 360: 263–268. 17586463 10.1016/j.bbrc.2007.06.043

[24] Kim KR, Yoshizaki T, Miyamori H, Hasegawa K, Horikawa T, et al (2000) Transformation of Madin-Darby canine kidney (MDCK) epithelial cells by Epstein-Barr virus latent membrane protein 1 (LMP1) induces expression of Ets1 and invasive growth. Oncogene 19: 1764–1771. 10777210 10.1038/sj.onc.1203502

[25] Kaykas A, Sugden B (2000) The amino-terminus and membrane-spanning domains of LMP-1 inhibit cell proliferation. Oncogene 19: 1400–1410. 10723131 10.1038/sj.onc.1203365

[26] Zeng Q, Tan YH, Hong W (1998) A single plasmid vector (pSTAR) mediating efficient tetracycline-induced gene expression. Anal Biochem 259: 187–194. 9618196 10.1006/abio.1998.2645

[27] Ndour PA, Brocqueville G, Ouk TS, Goormachtigh G, Morales O, et al (2012) Inhibition of latent membrane protein 1 impairs the growth and tumorigenesis of latency II Epstein-Barr virus-transformed T cells. J Virol 86: 3934–3943. 22258264 10.1128/JVI.05747-11 PMC3302486

[28] Horikawa T, Yang J, Kondo S, Yoshizaki T, Joab I, et al (2007) Twist and epithelial-mesenchymal transition are induced by the EBV oncoprotein latent membrane protein 1 and are associated with metastatic nasopharyngeal carcinoma. Cancer Res 67: 1970–1978. 17332324 10.1158/0008-5472.CAN-06-3933

[29] Horikawa T, Yoshizaki T, Kondo S, Furukawa M, Kaizaki Y, et al (2011) Epstein-Barr Virus latent membrane protein 1 induces Snail and epithelial-mesenchymal transition in metastatic nasopharyngeal carcinoma. Br J Cancer 104: 1160–1167. 21386845 10.1038/bjc.2011.38 PMC3068490

[30] Adriaenssens E, Mougel A, Goormachtigh G, Loing E, Fafeur V, et al (2004) A novel dominant-negative mutant form of Epstein-Barr virus latent membrane protein-1 (LMP1) selectively and differentially impairs LMP1 and TNF signaling pathways. Oncogene 23: 2681–2693. 14767477 10.1038/sj.onc.1207432

[31] Goormachtigh G, Ouk TS, Mougel A, Tranchand-Bunel D, Masy E, et al (2006) Autoactivation of the Epstein-Barr virus oncogenic protein LMP1 during type II latency through opposite roles of the NF-kappaB and JNK signaling pathways. J Virol 80: 7382–7393. 16840319 10.1128/JVI.02052-05 PMC1563735

[32] Bornkamm GW, Berens C, Kuklik-Roos C, Bechet JM, Laux G, et al (2005) Stringent doxycycline-dependent control of gene activities using an episomal one-vector system. Nucleic Acids Res 33: e137. 16147984 10.1093/nar/gni137 PMC1201338

[33] Ndour PA, Ouk TS, Brocqueville G, Mougel A, Vanhecke E, et al (2010) Inhibition of tumor necrosis factor-induced phenotypes by short intracellular versions of latent membrane protein-1. Cell Signal 22: 303–313. 19796681 10.1016/j.cellsig.2009.09.037

