# Supplementary figures and images for: LMP1-Induced Cell Death May Contribute to the Emergency of Its Oncogenic Property
Source: PLoS One. 2013 Apr 23;8(4):e60743. doi: 10.1371/journal.pone.0060743 (PMC3634045; doi:10.1371/journal.pone.0060743)

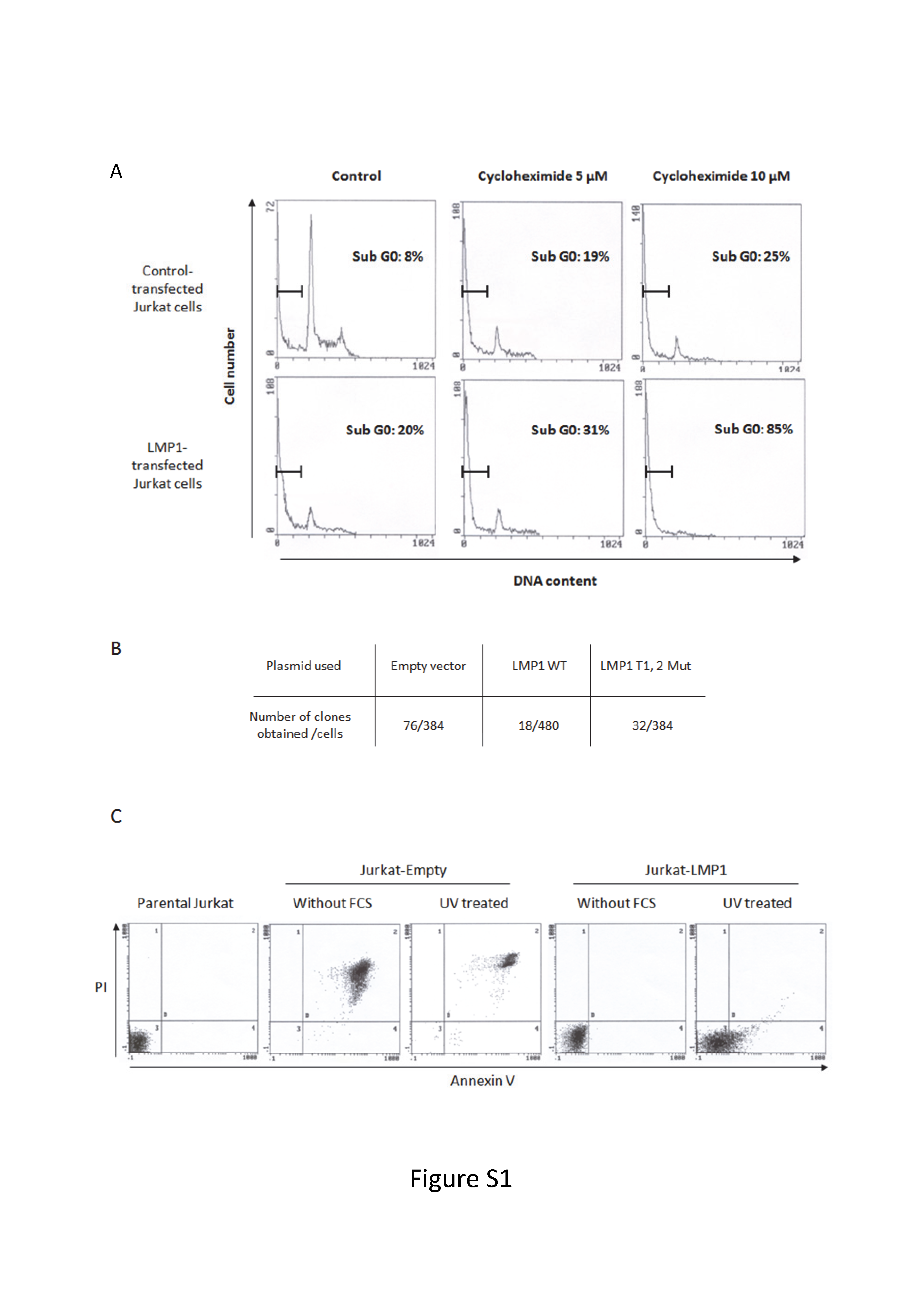

Supplement: Figure S1 — Ectopic expression of LMP1 in Jurkat cells induced both cell death and cell survival. A) Cell cycle analysis was performed after transient LMP1 transfection and cycloheximide treatment. Cells were transfected with empty vector as control or LMP1 encoding vector. After 24 h, cells were treated with the indicated doses of cycloheximide during 24 h and cell cycle was analyzed by flow cytometry. Percentage of sub G0/G1 cells was indicated. B) Number of clones obtained after stable transfection of LMP1-derived plasmids. After transfection, selection was added and cells were dispatched in 96-well plates. C) LMP1 protects Jurkat cells during serum deprivation or after UV irradiation. Stably transfected cells were starved during 24 h or treated by UV and cell death was monitored after annexin V and propidium iodine labeling. See File S1 for information. (TIF) [file pone.0060743.s001.tif]
