# Supplementary material for: LMP1-Induced Cell Death May Contribute to the Emergency of Its Oncogenic Property
Source: PLoS One. 2013 Apr 23;8(4):e60743. doi: 10.1371/journal.pone.0060743 (PMC3634045; doi:10.1371/journal.pone.0060743)
Supplement: File S1 — (DOCX) [file pone.0060743.s002.docx]

**File S1**

To generalize results obtained with the MDCK and HEK-293 cell lines, we investigated the effects of LMP1 in Hela and Jurkat cells. After transient transfection in Hela or Jurkat cells, cell death was monitored. In addition, we tried to obtain cells stably expressing LMP1 and number of clones was evaluated. In cells stably expressing LMP1, we measured the protective effect of LMP1.

**Methods**

*Reagent*

Cycloheximide was purchased from Sigma.

*Transfections*

Jurkat T cell line was transfected by Nucleofection using “Amaxa® Cell Line Nucleofector® Kit V” and X-001 program according to manufacturer (Amaxa, Lonza). The Hela cells were transfected as HEK 293 cell line.

*Measurement of cell death using flow cytometry analysis*

*i) Cell cycle*. Cell death was assessed using flow-cytrometry analysis after propidium iodide (PI, Sigma) labelling. For cell cycle analysis, cells were washed with ice‑cold PBS, and then incubated with ice-cold 70% ethanol. Fixed cells were washed and then treated with RNase A (1 mg/ml) and stained with PI (100 µg/ml). The percentage of cells in each phase of cell cycle (i.e G0/G1, S, G2M) as well as the percentage of dead cells (sub G0/G1) were then determined by measurement of DNA content using a flow cytometer (Coulter EPICS XL-MCL).

*ii) Annexin V detection*. Total cell death was measured using 50 µg/ml propidium iodide (PI) (Sigma) labeling, and apoptotic cells were assessed by Annexin V-phycoerythrin (PE) (BD Pharmingen) labeling. Briefly, 24 h after transfection of a LMP1 or control vector, cells were treated (24 h of serum starvation, or UVB irradiation). For UVB irradiation, we used the SpectroLinker XL-1000 UV crosslinker (Spectronics Corporation) with a wavelength of 312 nm and a power of 20 W/m^2^. Then, cells were washed and stained with Annexin V-PE according to the manufacturer’s instructions. Cell viability was assessed by addition of 50 µg/ml PI to samples just before flow cytometry analysis.

*Colony-formation assay*

After transfection, Jurkat cells were placed in fresh medium. Antibiotic was added after 24h and cells were dispatched in 96-well plates. The number of clones obtained was monitored.

For Hela cells, we used the same strategy than for HEK-293 cells.

**Results and Discussion.**

We generalize the results obtained by using two other cell lines, Hela and Jurkat. We have transfected a LMP1-encoding vector followed by selection of cells stably expressing LMP1 protein. With Hela cells, we obtained three times more clones, after transfection with empty vector compared to LMP1-transfected cells. In Jurkat, the number of clones obtained by limiting dilution was indicated in Supplemental Figure 1B. We obtained 76/384 viable clones (four 96 well plates were used) after transfection with empty vector, 18/480 viable clones after tranfection with LMP1-encoding vector, and 32/384 clones after transfection with LMP1 T1, 2Mut-encoding vector. These results confirm that LMP1 induces cell death after ectopic expression in all cells tested. To assess this cell death, we performed after transient experiment a measurement of cell death using flow cytometry analysis. After LMP1 or control vector transfection we realized a cell cycle analysis by flow cytometry (Supplemental Fig 1A). The percentage of cells in sub G0/G1 (loss of DNA content) is indicative of dead cells. Empty vector transfected cells exhibited 8% of cell death whereas LMP1 expression induced cell death (20%). Increasing doses of cycloheximide induced cell death in a dose-dependent manner: in control-transfected cells, cell death reached 25%, whereas in LMP1-transfected cells, 85% of the cells die. LMP1 induces cell death *per se* and sensitizes cells to chemotherapeutic agent-induced apoptosis. By contrast, after obtention of cell lines stably expressing LMP1, LMP1 protect jurkat cells of death due to serum starvation or UV radiation (Supplemental Fig 1C) according to the work of Kawanishi (1997).

**Figure legends**

**Figure S1. Ectopic expression of LMP1 in Jurkat cells induced both cell death and cell survival.**

**A)** Cell cycle analysis was performed after transient LMP1 transfection and cycloheximide treatment. Cells were transfected with empty vector as control or LMP1 encoding vector. After 24h, cells were treated with the indicated doses of cycloheximide during 24h and cell cycle was analyzed by flow cytometry. Percentage of sub G0/G1 cells was indicated. **B)** Number of clones obtained after stable transfection of LMP1-derived plasmids. After transfection, selection was added and cells were dispatched in 96-well plates. **C)** LMP1 protects Jurkat cells during serum deprivation or after UV irradiation. Stably transfected cells were starved during 24h or treated by UV and cell death was monitored after annexin V and propidium iodine labeling.
